# Unclear associations between small pelagic fish and jellyfish in several major marine ecosystems

**DOI:** 10.1038/s41598-019-39351-7

**Published:** 2019-02-28

**Authors:** Anders Frugård Opdal, Richard D. Brodeur, Kristin Cieciel, Georgi M. Daskalov, Vesselina Mihneva, James J. Ruzicka, Hans M. Verheye, Dag L. Aksnes

**Affiliations:** 1 Department of Biological Sciences, University of Bergen, Bergen, Norway; 2 Fish Ecology Division, Northwest Fisheries Science Center, National Oceanic and Atmospheric Administration, Newport, OR USA; 3 Auke Bay Laboratory, Alaska Fisheries Science Center, National Oceanic and Atmospheric Administration, Juneau, AK USA; 4 Institute of Biodiversity and Ecosystem Research, Sofia, Bulgaria; 5 Institute of Fish Resources, Varna, Bulgaria; 6 Cooperative Institute for Marine Resources Studies, Oregon State University, Newport, Oregon, USA; 7 Oceans and Coastal Research, Department of Environmental Affairs, Cape Town, South Africa; 8 Marine Research Institute, University of Cape Town, Cape Town, South Africa

## Abstract

During the last 20 years, a series of studies has suggested trends of increasing jellyfish (Cnidaria and Ctenophora) biomass in several major ecosystems worldwide. Some of these systems have been heavily fished, causing a decline among their historically dominant small pelagic fish stocks, or have experienced environmental shifts favouring jellyfish proliferation. Apparent reduction in fish abundance alongside increasing jellyfish abundance has led to hypotheses suggesting that jellyfish in these areas could be replacing small planktivorous fish through resource competition and/or through predation on early life stages of fish. In this study, we test these hypotheses using extended and published data of jellyfish, small pelagic fish and crustacean zooplankton biomass from four major ecosystems within the period of 1960 to 2014: the Southeastern Bering Sea, the Black Sea, the Northern California Current and the Northern Benguela. Except for a negative association between jellyfish and crustacean zooplankton in the Black Sea, we found no evidence of jellyfish biomass being related to the biomass of small pelagic fish nor to a common crustacean zooplankton resource. Calculations of the energy requirements of small pelagic fish and jellyfish stocks in the most recent years suggest that fish predation on crustacean zooplankton is 2–30 times higher than jellyfish predation, depending on ecosystem. However, compared with available historical data in the Southeastern Bering Sea and the Black Sea, it is evident that jellyfish have increased their share of the common resource, and that jellyfish can account for up to 30% of the combined fish-jellyfish energy consumption. We conclude that the best available time-series data do not suggest that jellyfish are outcompeting, or have replaced, small pelagic fish on a regional scale in any of the four investigated ecosystems. However, further clarification of the role of jellyfish requires higher-resolution spatial, temporal and taxonomic sampling of the pelagic community.

## Introduction

Increases in cnidarian and ctenophore (hereafter collectively termed jellyfish) biomasses have been reported for a number of major ecosystems^1–4^, and various qualitative reviews^5–7^ and meta-analyses^8^ suggest numerous localized increases worldwide. However, global data are sparse and susceptible to the alternative interpretations of no trend^9,10^, long-term cyclical variation^11^ or publishing and citation biases^12,13^.

Based on observed dietary overlap (i.e., crustacean zooplankton prey) between small (zooplanktivorous) pelagic fish and jellyfish^14–16^ it has been proposed that when pelagic fish stocks have been reduced, either through fishing, climatic conditions or other disturbances, jellyfish may become a significant competitor^1,2,17,18^, and in turn, functionally replace small pelagic fish^7,19^. Moreover, experimental and field evidence indicating that jellyfish are efficient predators upon early life stages of fish^20–23^ has further suggested that jellyfish predation may constrain recruitment of small pelagic fish stocks^2,7,19^.

According to the above studies, three non-mutually exclusive interpretations or observations can be stated: (1) jellyfish have functionally replaced small pelagic fish, and consume a larger part of the common resource than small pelagic fish, (2) jellyfish and small pelagic fish are competing for a limited resource, and (3) jellyfish are significant predators upon early life stages of small pelagic fish, and consequently constrain recruitment of the latter. For each of these statements, we have formulated a hypothesis. These were tested using the best available time-series data of small pelagic fish, jellyfish and crustacean zooplankton biomass for three major ecosystems: the Southeastern Bering Sea, the Northern California Current, and the Black Sea (Supplementary Fig. S1a–c). In addition, we also included the Northern Benguela Current (Supplementary Fig. S1d) for which data only allowed to investigate whether jellyfish have functionally replaced small pelagic fish, and are the dominant predators of a common resource. All four systems have been characterized by high, or periods of high, jellyfish biomass, and concerns have been raised regarding the potential impact of jellyfish on small pelagic fish. Jellyfish in these areas have been suggested to be important competitors of various forage fishes in the Southeastern Bering Sea^18,24^ and the Northern California Current^3,25–27^, of sardines in the Northern Benguela^1,17^ and of anchovy in the Black Sea^2,28,29^.

**Figure 1:**
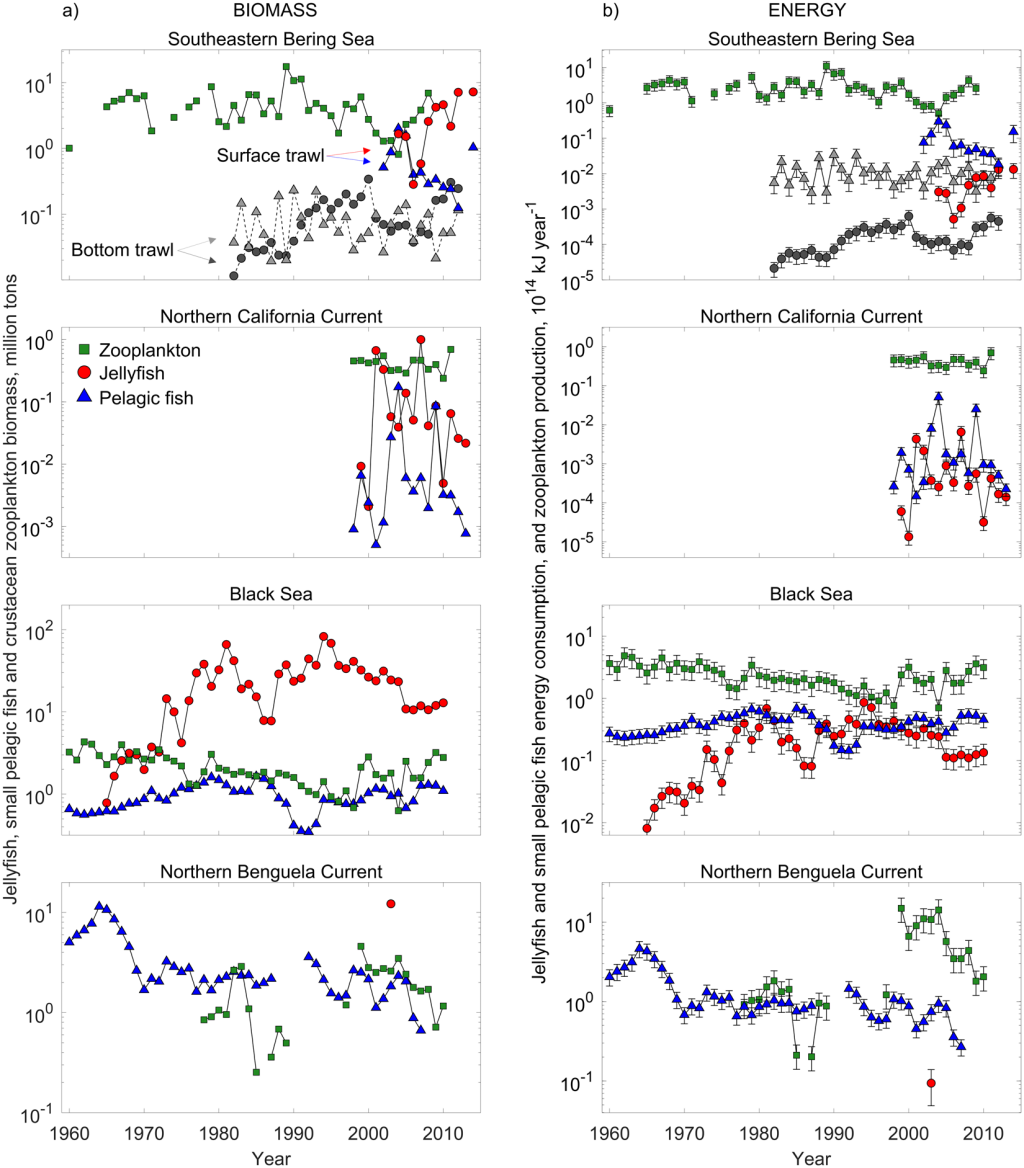
Biomass and corresponding energy requirements in four ecosystems. The left panels (**a**) show biomass time-series of jellyfish (circles), small pelagic fish (triangles) and crustacean zooplankton (squares) in four ecosystems, updated from previously published studies (see Supplementary Table S1). The right panels (**b**) show the corresponding estimates of energy consumption rates by jellyfish and small pelagic fish compared with estimated crustacean zooplankton production rates. For jellyfish and pelagic fish, vertical bars denote the 95% confidence intervals associated with parameter uncertainty in estimating energy consumption (*E*, Table 4) and the mean individual weight range (*M*_*ind*_, Table 4). For crustacean zooplankton production, vertical bars represent the 95% confidence intervals around a normally distributed caloric density range (*p*_*cal*_), as well as uncertainty in the area-specific *PB* ratios (Table 4). For the Southeastern Bering Sea, small pelagic fish and jellyfish estimates from two independent surveys are shown. A bottom trawl survey (RACE; 1982–2012, grey symbols and hatched lines), and a surface trawl survey (BASIS, 2002–2013, coloured symbols and solid lines).

Based on general ecological theory^30,31^ three *a priori* hypotheses were formulated in accordance with the above statements: H1) jellyfish consume a larger part of the common resource than small pelagic fish, H2) there is a negative correlation between jellyfish and small pelagic fish biomass, or between jellyfish biomass and the common resource, and H3) there is a negative correlation between jellyfish biomass and small pelagic fish recruitment.

## Results

In terms of biomass (Fig. 1a) it is evident that both small pelagic fish and jellyfish exhibit large fluctuations in all systems across the available sampling years. However, in some systems there are also some temporal log-linear trends in biomass, including the increase of jellyfish in the Southeastern Bering Sea (~5500 tons year ^−1^, *p*-value = 0.01) and the decrease of small pelagic fish in the Northern Benguela (~80.000 tons year^−1^, *p*-value < 0.01).

### Comparison of energy consumption between fish and jellyfish (H1)

With regards to hypothesis H1 - *jellyfish consume a larger part of the common resource than small pelagic fish*, the estimated energy requirements (*E*) for the *recent* time-periods (from 2000 onwards) suggest that small pelagic fish require on average 2–30 times more energy per year compared with their respective jellyfish competitors, depending on ecosystem (Fig. 1b and Table 1).

**Table 1:** Total jellyfish and small pelagic fish energy requirements, the relative energy requirement of each, and estimated crustacean zooplankton production rates for the four ecosystems.

System and years		Mean energy requirement, pelagic fish + jellyfish		Jelly-fish	Pelagic fish	Mean zooplankton production	
	n	10^12^ kJ year^−1^	SD	%	%	10^12^ kJ year^−1^	SD
**Bering Sea**							
2004–2009 BASIS	6	13	9	9	91	213	128
1982–2009 RACE	28	1.2	0.86	2	98	290	220
≤1991 *before*	10	1.4	1.1	1	99	441	291
>1991 *after*	18	1.1	0.72	3	97	206	104
≥2000 *recent*	10	1.1	0.57	3	97	171	112
**NCC**							
1999–2013	14	0.74	1.4	31	69	42	11
**Black Sea**							
1965–2010	46	64	22	33	67	222	87
≤1976 *before*	12	42	11	12	88	303	75
>1976 *after*	34	72	20	41	59	193	72
≥2000 *recent*	11	62	12	28	72	230	85
**N. Benguela**							
2003	1	83		11	89	11	

For the Southeastern Bering Sea, two independent surveys are available: a bottom trawl survey (RACE, 1982–2012) and a surface trawl survey (BASIS, 2004–2009). For comparison, the two longest time-series, the Southeastern Bering Sea (RACE) and the Black Sea, have been divided into three time-periods; two periods showing results before and after the historical increase in jellyfish, and one period defining the most recent years (≥2000). SD = standard deviation and NCC = Northern California Current.

For the Southeastern Bering Sea (RACE bottom trawl survey, 1982–2012) and the Black Sea, where data were also available *before* the apparent increases in jellyfish biomass (see Methods), it is evident that both the biomass and energy requirements of the jellyfish have increased relative to those of small pelagic fish (Fig. 1b and Table 1). For instance, while jellyfish in the Black Sea accounted for ca. 12% of the combined fish-jellyfish energy consumption before 1976 (when jellyfish biomass started to increase), they accounted on average for ca. 42% in the period *after* (1977–2010). However, this appears to have declined somewhat in the *recent* time-period (2000–2010) to ca. 30%. This last estimate is comparable to that for the Northern California Current, where jellyfish were estimated to account for ca. 31% of the combined fish-jellyfish energy consumption (1999–2013).

### Associations between small pelagic fish, jellyfish and crustacean zooplankton (H2 and H3)

**Figure 2:**
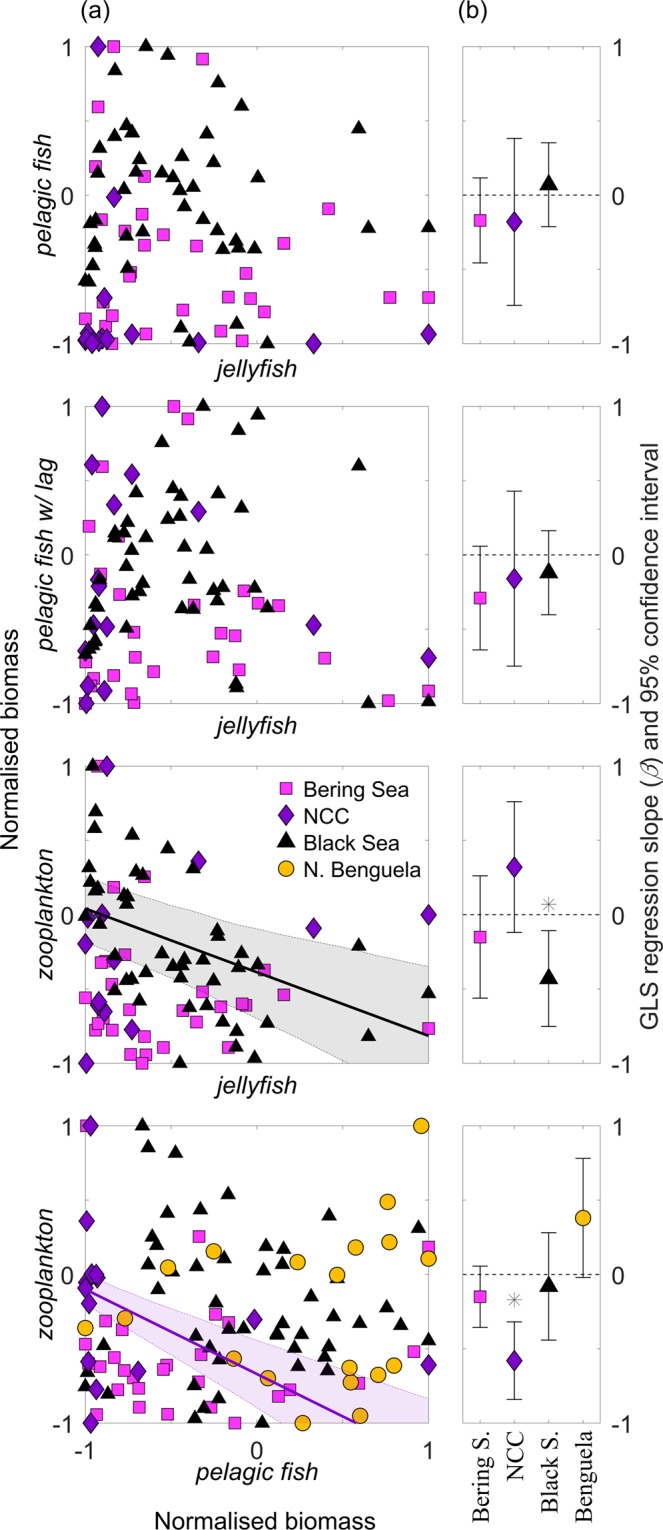
Graphical (**a**) and statistical (**b**) relationships between jellyfish, small pelagic fish and crustacean zooplankton biomass for four ecosystems; the Southeastern Bering Sea (pink squares), the Northern California Current (NCC, purple diamonds), the Black Sea (black triangles) and the Northern Benguela Current (orange circles). The left panels (**a**) show the graphical relationships between biomasses (normalised between -1 and 1) over time, while the right panels (**b**) show the generalised least squares (GLS) regression slope (*β*) with 95% confidence intervals corrected for 1^st^ order autoregressive processes (see Methods). For the association *jellyfish~pelagic fish w/lag* (to test lagged effects of jellyfish associated with constrained fish recruitment – see Methods), results are shown for the lag with the best negative fit (see also Table 2). All *p*-values are listed in Table 2. In the left panels, lines represent regression slopes (*β)* significantly different from zero (GLS, *p-values* < 0.05), denoted by * in the right panels (**b**) Shading denote the 95% confidence interval of the regression. Regression line-colour and shading refers to that of the corresponding area.

Testing for a negative *pelagic fish* ~ *jellyfish* association (all study-areas except N. Benguela), no statistically significant relationships were found within study-areas (all *p*-values > 0.42; Table 2 and Fig. 2b), nor in the combined models (all *p*-values > 0.41 for regression slope *β*, Table 3). Testing for a negative *zooplankton ~ jellyfish* association (all study-areas except N. Benguela), we found a significant negative regression for the Black Sea (*p*-value < 0.01, Table 2 and Fig. 2b), but no significant effects in the combined models (all *p*-values > 0.18, Table 3).

**Table 2:** Results from the generalised least squares (GLS) regression model (*m*_*A*_) between time-series of jellyfish, small pelagic fish and crustacean zooplankton biomass for four ecosystems.

Associations and system	*pelagic fish~ jellyfish*			with 1–3 year time-lag (best negative fit shown)				*zooplankton~ jellyfish*			*zooplankton~ pelagic fish*		
	*n*	*β*	*p*-val	*n*	*lag*	*β*	*p*-val	*n*	*β*	*p*-val	*n*	*β*	*p*-val
**Bering Sea**													
BASIS 2002-	10	0.007	0.98	8	1	−0.21	0.57	6	0.27	0.58	8	−0.35	0.29
RACE 1982-	31	−0.17	0.22	28	2	−0.29	0.10	28	−0.15	0.44	28	−0.15	0.15
**NCC**	15	−0.18	0.5	14	1	−0.16	0.57	13	0.32	0.14	14	**−0**.**58**	**<0**.**01**
**Black Sea**	46	0.07	0.61	43	3	−0.12	0.41	**46**	**−0**.**43**	**<0**.**01**	51	−0.08	0.63
**N. Benguela**											19	0.38	0.06

All regressions are corrected for 1^st^ order autoregressive processes. To analyse potential effects of jellyfish on small pelagic fish recruitment, the association between jellyfish and small pelagic fish biomass was analysed with 1–3 year lag of fish biomass. For the Southeastern Bering Sea, two independent surveys are available: a bottom trawl survey (RACE, 1982–2012) and a surface trawl survey (BASIS, 2004–2009). Significant regression slopes (*β*) are denoted in bold (*p*-value < 0.05). NCC = Northern California Current.

**Table 3:** Results from the generalised least squares (GLS) regression models (*m*_*1*_*-m*_3_) between time-series of jellyfish, small pelagic fish and crustacean zooplankton biomass analysed across four ecosystems (*A*).

Associations and model	*pelagic fish ~ jellyfish (n = 92)*			*zooplankton ~ jellyfish (n = 87)*			*zooplankton ~ pelagic fish (n = 112)*		
	AIC	*β*	*p*-value	AIC	*β*	*p*-value	AIC	*β*	*p*-value
*m*_*0*_, Y ~ 1	149.5			105.4			133.5		
*m*_*1*_, Y ~ α + βX	151.5	−0.03	0.83	106.4	−0.11	0.3	135.5	−0.004	0.95
*m*_*2*_, Y ~ α + βX + cA	141.6	−0.1	0.41	104.9	−0.14	0.18	136.7	−0.01	0.88
*m*_*3*_, Y ~ α + βX + cA + dXA	145.5			102.2			137.9		
Bering Sea		−0.14	0.48		−0.18	0.36		**−0**.**15**	**0**.**03**
NCC		−0.09	0.87		0.17	0.19		0.04	0.27
Black Sea		−0.05	0.75		−0.48	0.23		−0.13	0.18
Northern Benguela								0.32	0.08

All models are corrected for 1st order autoregressive processes. The Akaike Information Criterion (AIC) is presented for the entire model, while only parameter β is presented with estimated value and *p*-value. For model *m*_*3*_, *β* estimates and *p*-values are denoted for each system separately. Significant regression slopes (*β*) are denoted in bold (*p*-value < 0.05), while best model fit regardless of statistical significance is denoted by underline. NCC = Northern California Current.

For the *zooplankton* ~ *pelagic fish* association (all study-areas), which is partly related to resource limitation, a significant negative regression was found for the Northern California Current (*p*-value < 0.01; Table 2 and Fig. 2). For the combined models, model *m*_3_ suggests a significant negative relationship in the Bering Sea (*p*-value = 0.03 for regression slope *β*, Table 3). However, model *m*_3_ is also found to be the least favourable model according to the AIC (*m*_3_, AIC = 137.9), which is significantly higher than that of the null-model (*m*_0_, AIC = 133.5, Table 3).

No significant negative regressions were found for the *pelagic fish ~ jellyfish* association with time lags of 1 to 3 years (*p*-values > 0.1; Table 2 and Fig. 2), relating to hypothesis H3. Similarly, we found no associations between harvest rates of small pelagic fish (Supplementary Fig. S2) and the biomass of jellyfish (*p*-values > 0.3).

The statistical power for the pairwise associations were in nearly all cases found to be low, given the data sample sizes (*n*) and estimated effect sizes (*R*) (Supplementary Table S2). This is in accordance with the small GLS regression coefficients (β) and the corresponding high *p*-values (Fig. 2 and Table 2). With the exception of the pairwise correlation *zooplankton ~ jellyfish* in the Black sea (*R* = 0.57, *power* = 0.99; Supplementary Table S2), all statistical power estimates were < 0.4, and most were < 0.2 (Supplementary Table S2), well below the desired minimum level of 0.8 as reported by Cohen^32^. This can be explained by the low sample sizes and/or the low effect sizes in the majority of the pairwise associations (*n* ≤ 31, *R* ≤ 0.27; Supplementary Table S2). To achieve a statistical power greater than 0.8 with an *R ≤ *0.27, the sample size (*n*) should be > 104. Similarly, assuming a sample size *n* ≤ 31, the effect size (*R*) should be > 0.48. In this study, no pairwise analysis had sample sizes n > 51, and only two had an effect sizes *R* > 0.57; *zooplankton ~ pelagic fish* in the Bering Sea (BASIS) and *zooplankton ~ jellyfish* in the Black Sea (Supplementary Table S2).

The result from the confirmatory factor analyses (CFA, Supplementary Table S3) did not provide convincing support for the structural equation model set up to test hypothesis H2 and H3 (Supplementary Fig. S3) in the three ecosystems (Bering Sea, Northern California Current and the Black Sea). Some support may be found for the model for the Northern California Current with regards to the exact-fit Chi-square test^33^, but fails to reject the approximate tests of poor model fit^34,35^ (Supplementary Table S3).

## Discussion

Except for the *jellyfish ~ zooplankton* association in the Black Sea, the data available cannot be used to support the three proposed hypotheses regarding competition between small pelagic fish and jellyfish in the four ecosystems studied here. Estimation and comparison of fish and jellyfish energy requirements suggest that, on average, the jellyfish populations have lower energy requirements than the small pelagic fish populations in each ecosystem over most of the time-periods investigated. This does not suggest that jellyfish consume a *larger* part of the common resource than small pelagic fish (H1). However, during some periods, the energy requirements of jellyfish can match or exceed those of small pelagic fish, but this does not appear to have led to a robust, long-lived replacement of their relative roles as consumers in any of the studied ecosystems. In addition, we note that in the Northern California Current and the Black Sea, jellyfish in the most recent time-period do account for around 30% of the combined fish-jellyfish energy consumption – which is significant.

We acknowledge the uncertainty associated with the use of respiration rates (E_R_, for basal metabolic costs) combined with production estimates (E_P_, for growth and reproduction costs) to calculate total consumption rates. Respiration experiments, for both fish and jellyfish, are known to underestimate oxygen consumption compared to natural conditions, since they are typically designed to remove the respiratory costs of movement, feeding and digestion^36–38^. Thus, neither of these costs are explicitly accounted for in this study. Also, it is suggested that jellyfish predation rates might in fact be higher than the actual feeding rate due to excess prey becoming entangled and killed in their tentacles^39^. However, it is not possible to conclude that the respiration rates used in this study are more biased for either fish or jellyfish. The respiration rates used here are, per body carbon, identical for both fish and jellyfish^40^.

The use of literature-derived values for production-to-biomass (PB) ratios, energy densities and carbon content also provide additional uncertainty. In particular, published PB ratios for jellyfish in the Southeastern Bering Sea and the Northern Benguela are uncertain as they are not measured entities, but estimates derived from ecosystem models^41,42^. However, the overall estimates of energy requirement do not appear to be particularly sensitive to the parameterisation of PB ratios. For instance, a 10-fold increase in the PB ratio of jellyfish in the Southeastern Bering Sea (from 0.88 to 8.8 year^−1^) results in a moderate increase in the mean share of energy consumption from 2% to 6% over the period 1982–2009. An equivalent increase in the PB ratio of jellyfish in the Northern Benguela (from 0.44 to 4.4 year^−1^) results in an increase from 11% to 13% of the energy share in the year 2000. Although parameter uncertainty has been incorporated in the analysis where this information was available in the literature, and otherwise assumed relative uncertainties (SD = 0.5·mean), the calculated energy requirements should be considered rough estimates.

A direct comparison of small pelagic fish and jellyfish biomasses and energy requirements should be undertaken with caution. Differences in catchability between fish species and jellyfish in ocean sampling surveys are considerable, both between (Fig. 1a, Southeastern Bering Sea) and within^43^ different sampling gear types. The estimates of biomass and energy requirements are sensitive to the different (and unknown) catchabilities of fish and jellyfish in each survey, as well as the seasonal fluctuations in biomass that are not captured in spring/summer surveys, such as in the Northern California Current and the Bering Sea. Specifically, Brodeur *et al*.^18^ reported that for the Bering Sea RACE survey (1982–2012), the bottom trawl used for sampling was likely to underestimate jellyfish abundance. When compared to the biomass estimates from the finer meshed surface trawl samples conducted in the BASIS survey (2004–2009), this discrepancy is evident for both jellyfish and forage fish (Fig. 1a). Thus, in terms of energy consumption during the period with overlapping surveys, small pelagic fish still consume more than ten times the energy of jellyfish, regardless of sampling methodology. A more general challenge is that certain groups of individually small and fragile gelatinous zooplankton, such as many ctenophore and hydromedusa species, are likely to be under-represented in data records since they can be extruded through the net with only moderate pressure, and if captured, are often unidentifiable. In any case, neither sampling nor parameter uncertainty should have biased the pairwise statistical analyses provided the usage of sampling gears and survey methodologies have remained consistent through time. This assumes that total biomasses are not severely underestimated, or that temporal increase or decrease in biomass does not occur in a particular size range that is consistently undersampled by the nets.

Except for the significant negative regression found for the *jellyfish ~zooplankton* association in the Black Sea, the lack of negative regressions between jellyfish and small pelagic fish biomasses, and between jellyfish and zooplankton biomasses does not provide support for hypothesis H2, that there is a negative correlation between jellyfish and small pelagic fish biomass, or between jellyfish biomass and the common resource. However, the negative *jellyfish ~ zooplankton* regression in the Black Sea, together with the negative *pelagic fish ~ zooplankton* regression in the Northern California Current might be indicative of crustacean zooplankton being a limited resource, although we did not find any relationship between harvest rates of small pelagic fish and the biomass of jellyfish, which could have indicated release from competition.

These findings are not consistent with earlier studies reporting negative correlations between small pelagic fish and jellyfish in the Southeastern Bering Sea^18,44^, Northern California Current^44^ and the Black Sea^29^. Regarding the Southeastern Bering Sea and the Black Sea, some of these discrepancies are likely explained by the addition of new data points. For the Southeastern Bering Sea^18^, this constitutes an additional 13 years, while for the Black Sea^29^ it includes all years after 1988 (23 additional years), which then also includes the ctenophore *M*. *leidyi* - not present prior to 1988^29^. Robinson *et al*.^44^ reported jellyfish-fish replacement cycles for both the Southeastern Bering Sea (1982–2012) and the Northern California Current (1998–2010). However, this cycling was inferred from visual interpretation of apparent trends in the biomass time-series, which might explain the discrepancy with our statistical analysis.

The investigated hypotheses considered small pelagic fish and jellyfish as aggregated functional groups. Individual species-to-species relations were not analysed, some of which may express negative correlations and some of which may have weak, absent or positive correlations. Within the Northern California Current, statistically significant negative correlations between individual species of small pelagic fish and jellyfish have been found within particular seasons^25^. Biomass of the dominant scyphozoan jellyfish (*Chrysaora fuscescens*) was inversely correlated with Pacific sardine (*Sardinops sagax*) and northern anchovy (*Engraulis mordax*) biomasses in June and September, but biomass of *C*. *fuscescens* was not correlated with that of Pacific herring (*Clupea pallasii*). Likewise, a negative correlation was found between adult salmon returns to the Columbia River and coastal *C*. *fuscescens* biomass during previous summers when salmon smolts first enter the ocean^45^. This could indicate that jellyfish may have local spatiotemporal effects on small pelagic fish, but that these effects are so insignificant to be statistically distinguishable from the overall inter-annual variability in the data.

Furthermore, it is possible that predation by other species unaccounted for, or low spatial and temporal precision in biomass estimates, may mask potential jellyfish effects (Type II error). The latter is evident when analysing the statistical power of the pairwise associations (Supplementary Table S2). Low observed effect sizes (*R* ≤ 0.27) require high sample size (*n* > 104), and low observed sample sizes (*n* ≤ 31) require high effect sizes (*R* ≥ 0.48) to achieve sufficient statistical power (≥0.8). Because all our analyses have sample sizes *n* ≤ 51 and most have effect sizes *R* ≤ 0.27, we cannot rule out the possibility of Type II errors.

Potential jellyfish predation on early life stages of fish^20,22,23^ is difficult to infer from the biomass time-series data used here. If such predation is proportional to jellyfish biomass, it is not unreasonable to expect a time-lagged response in the small pelagic fish biomass as a consequence of jellyfish predation on fish eggs and larvae. The lagged GLS model did not reveal any such negative correlations (H3). However, strong predation effects from jellyfish on fish eggs and larvae have generally been found in relatively confined areas such as bays or fjords^15,22,46,47^, and thus might be difficult to observe at the scale of ecosystems spanning large areas of open water. Fish recruitment is also known to be a highly unpredictable process with several potential drivers^48–51^. Even if jellyfish has had an effect on recruitment of small pelagic fish, it might not be sufficiently strong to allow it to be disentangled from other factors.

Regarding the hypotheses H2 and H3, it is evident that a pairwise correlation analysis is a somewhat simplistic approach towards dealing with potentially complex predator-prey relationships. However, the addressed statements relating to the negative consequences of jellyfish increase are also simple in their original formulations^1,2,7,17,19,24^, and the expected inverse proportional change between neighbouring trophic levels is in line with general ecological theory^30,31^. Also, increasing model complexity to include multiple hypotheses (H2 and H3) in a structural equation framework with a confirmatory factor analysis, failed to find convincing support for hypothesis H2 and H3.

To conclude, we find that the best available time-series data do not provide evidence that jellyfish as a functional group are outcompeting, or have replaced, small pelagic fish as a functional group in any of the four investigated ecosystems. It is clear that the relatively large uncertainties in the data available may obscure associations that could have been detected with data sets of higher quality. However, strong statements regarding the relationship between small pelagic fish and jellyfish should also be supported with data. Thus, the outcome of the tests we have performed are relevant to the best available data in hand. Further clarification of the role of jellyfish requires improved sampling, particularly of jellyfish species, and higher-resolution spatial and temporal sampling of pelagic community compositions.

## Materials and Methods

### Biomass time-series

Annual mean biomass estimates of jellyfish, small pelagic fish and crustacean zooplankton (assumed common resource) for all four ecosystems (Fig. 2a) were compiled from a range of published sources (Supplementary Table S1 and Fig. S1). Note that in the Southeastern Bering Sea, jellyfish and small pelagic fish were sampled at the same stations during summer in two different programs: the BASIS surface trawl survey 2002–2014 (jellyfish sampled from 2004), and the RACE bottom trawl survey for the period 1982–2014. These programs were analysed separately.

### Energy consumption and production estimates (H1)

The hypothesis H1 - *jellyfish consume a larger part of the common resource than small pelagic fish*, was tested by comparing the yearly energy consumption of small pelagic fish and jellyfish that were estimated from biomass and metabolic rates^40^. Crustacean zooplankton biomasses together with caloric densities and daily crustacean zooplankton production rates were used to estimate the common prey resource available to planktivorous fish and jellyfish.

Annual energy consumption rates (*E*, J year^−1^) for jellyfish and small pelagic fish populations were estimated as the sum of energy costs (J year^−1^) for respiration (E_*R*_) and production (*E*_*P*_), *E* = *E*_*R*_ + *E*_*P*_. Due to a lack of data, the additional energy costs associated with defecation and excretion were not accounted for, and thus were assumed to be similar per unit carbon for both fish and jellyfish. Annual energy costs of respiration (*E*_*R*_, year^−1^) for small pelagic fish and jellyfish populations were defined as:

1 $${E}_{R}=R\cdot {k}_{1}\cdot \frac{BM}{{M}_{ind}}\cdot 365,$$

where *R* is the respiration rate of an individual (mmol O_2_ ind^−1^ d^−1^), and *k*_1_ (kJ mmol O_2_^−1^, Table 4) converts from mmol O_2_ to Joules. *BM* is population biomass (g wet weight) and *M*_*ind*_ is individual wet weight (g, Table 4). Energy costs of production (*E*_*P*_, year^−1^) for small pelagic fish and jellyfish populations were estimated from production to biomass ratios (*PB* year^−1^, C), specified for each ecosystem (Table 4):

2 $${E}_{P}=BM\cdot {\rho }_{C}\cdot PB\cdot {k}_{2},$$

where *ρ*_*C*_ is the carbon density (g C g wet weight^−1^) specified for fish and jellyfish, and *k*_2_ converts from organic carbon to Joules (kJ g organic carbon^−1^; Table 4).

**Table 4:** Description, values and units for parameters used to calculate yearly small pelagic fish and jellyfish energy requirements and crustacean zooplankton production.

Description	Abbrev.	Value and unit	Reference
**Total area**	***A***	**m** ^**2**^	
Bering Sea (survey area)		1.0·10^11^ m^2^	^62^
NCC (survey area)		3.7·10^10^ m^2^	^26^
Black Sea (productive area)		2.8·10^11^ m^2^	^2^
Northern Benguela		8.6·10^10^ m^2^	^1^
**Energy requirement**	***E***_***P***_, ***E***_***R***_	**J day** ^**−1**^	
Weight (fish, jellyfish)	*M*_*ind*_	g	
Bering Sea, mean ± SD		130 ± 94^a^, 376 ± 141	^18, 63^
NCC, mean ± SD		94 ± 37^b^, 298 ± 83	^64– 66^
Black Sea, mean ± SD		10 ± 3.5^c^, 14 ± 7^d^	^16, 67, 68^
N. Benguela, mean ± SD		84 ± 20^e^, 60 ± 15	^69, 70^
Conv., mmol O_2_ to Joules	*k*_1_	0.42 kJ mmol O_2_^−1^	(k_1_ = *a* · *b*)
Conv., mmol to g O_2_	*a*	0.031 g mmol O_2_^−1^	^71^
Conv., g O_2_ to Joules	*b*	13.56 kJ g O_2_^−1^)	^72^
Carbon density	*ρ*_*c*_	g C g WW^−1^	
Pelagic fish, mean ± SD		0.117 ± 0.05 g C g WW^−1^	^73^
Jellyfish, mean ± SD		0.008 ± 0.004 g C g WW^−1^	^74^
Prod: biomass (fish, jellyfish)	*PB*_*f*_, *PB*_*j*_	year^−1^	
Bering Sea, mean ± SD*		0.8 ± 0.4, 0.88 ± 0.44 year^−1^	^41^
NCC, mean ± SD*		2.0 ± 1.0, 7.5 ± 3.8 year^−1^	^75^
Black Sea, mean ± SD*		1.53 ± 0.8, 11 ± 5.5 year^−1^	^76^
N. Benguela, mean ± SD*		1.35 ± 0.7, 0.44 ± 0.22 year^−1^	^42^
Conv., carbon to Joules	*k*_2_	46 kJ g C^−1^	^77^
**Respiration**	***R***	**mmol O**_**2**_ **ind** ^**−1**^ **d** ^**−1**^	
Activation energy	*E*_*a*_	0.65 eV	
Boltzmann’s constant	*k*	8.62·10^−5^ eV K^−1^	
Mean abs. temperature ± SD	*T*	K	
Bering Sea		278 ± 2.2 K (3.8 °C)	^53^
NCC		283 ± 1.6 K (10.1 °C)	^53^
Black Sea		284 ± 2.9 K (10.7 °C)	^53^
N. Benguela		289 ± 1.6 K (16.2 °C)	^53^
Scaling constant	*B*_0_	1 (no unit)	^40^
Fish parameter, *b* ± SE	*b*_*f*_	0.781 ± 0.014	^40^
Jellyfish parameter, *b* ± SE	*b*_*j*_	0.780 ± 0.01	^40^
**Zooplankton production**	***P***	**J year** ^**−1**^	
Caloric density of calanoids	*ρ*_*cal*_	238–1230 cal g^−1^	^78^
Prod: biomass	*PB*_*zoo*_	day^−1^	
Bering Sea, mean ± SD*		0.055 ± 0.028 day^−1^	^79^
NCC, mean ± SD*		0.09 ± 0.045 day^−1^	^80^
Black Sea mean ± SD*		0.1 ± 0.5 day^−1^	^81^
Conv., calories to Joules	*k*_3_	4.18 J cal^−1^	^71^

For parameters where uncertainties are not available in the literature, standard deviation (SD) is assumed to be 50% of the mean (denoted with *).

NCC = Northern California Current and WW = wet weight. Denoted comments: ^a^based on herring, ^b^based on a weighted relationship between sardine [~146 g] and anchovy [~15 g], ^c^based on anchovy, ^d^based on the weighted relationship between of *A*. *aurita* [~34 g] and *M*. *leidyi* [~5 g], ^e^based on sardine.

Individual respiration rates (*R*, mmol O_2_ ind^−1^ d^−1^) were calculated from log-linear relationships with body wet weight (M_ind_, g ind^−1^)^40^:

3 $$R={B}_{0}\cdot \,{e}^{\frac{-{E}_{a}}{kT}}\cdot {M}_{ind}^{b},$$

where *B*_*o*_ is a scaling constant and ${e}^{\frac{-{E}_{a}}{kT}}$ is a temperature standardization term, of which *E*_*a*_ is the activation energy (eV), *k* is the Boltzmann’s constant (eV K^−1^), and *T* is absolute temperature (*K*). For temperature, annual means and standard deviations for each area were extracted from the Levitus climatology World Ocean Atlas 1998^52,53^. All parameter values and associated uncertainties are listed in Table 4.

For the purpose of comparison, and to obtain a reference estimate of the potential resource availability, the annual mean energy production rate of crustacean zooplankton (*P*_*zoo*_, J year^−1^) was also estimated:

4 $${P}_{{zoo}}=B{M}_{{zoo}}\cdot {\rho }_{{cal}}\cdot P{B}_{{zoo}}\cdot {k}_{3}\cdot 365,$$

where *BM*_*zoo*_ is the crustacean zooplankton population biomass (g), *ρ*_cal_ is the caloric density (cal g^−1^), *PB*_*zoo*_ is daily production to biomass ratio (d^−1^, C) and k_3_ is used to convert calories to Joules (J cal^−1^) (Table 4). For the Northern Benguela Current, the daily mean crustacean zooplankton production rate (equivalent to *BM*_*zoo*_*·PB*_*zoo*_ in equation 4) was derived by averaging summed species-specific production rates per sample for all samples collected in a given year^54^. Production rates of calanoid species were calculated from the species- and stage-specific body masses^54^ and their respective size-specific daily growth rates^55^ in the Northern Benguela Current. Production rates of cyclopoid species were estimated according to Huggett *et al*.^56^.

For all above calculations, parameter uncertainty (standard deviation of the mean) was incorporated either directly from literature values, or if unavailable, was assumed to be 50% of the mean parameter value (Table 4).

Population mean energy consumption rates of jellyfish and small pelagic fish and population mean production rates of crustacean zooplankton were averaged and compared over multi-annual time-periods. For the two longest time-series (the Southeastern Bering Sea RACE survey and the Black Sea), average rates were calculated for three time-periods; *before*, representing a period prior to jellyfish biomass increase, and *after*, representing the period following jellyfish biomass increase. A simple change point analysis (*findchangepts)*, Matlab R2016b)^57^ was applied to objectively find the two periods with the largest difference in jellyfish biomass. For the Southeastern Bering Sea (RACE bottom trawl survey) these periods were 1982–1991 (*before*) and 1992–2009 (*after*), and for the Black Sea they were 1965–1976 (*before*) and 1977–2010 (*after*). In addition, a third time-period was defined to represent the most recently collected data, named *recent*, and set to the years ≥ 2000. For the shorter time-series, the Southeastern Bering Sea BASIS survey (2004–2009), the Northern California Current (1999–2013) and the Northern Benguela Current (2003), all years were considered *recent*.

### Associations between small pelagic fish, jellyfish and crustacean zooplankton (H2 and H3)

Hypothesis H2 - there is a negative correlation between jellyfish and small pelagic fish biomass, or between jellyfish biomass and the common resource, and H3- there is a negative correlation between jellyfish biomass and small pelagic fish recruitment were tested through a series of pairwise associations within and across ecosystems. A 1–3 year lag for small pelagic fish biomass was used as a proxy for delayed effects of jellyfish predation on fish recruitment. Lag time is expected to be dependent on the age at which recruits are caught by fishery or sampling gear, which is less than 3 years for all species and ecosystems investigated.

Although not directly related to the above hypotheses, we also examined if there was a positive relationship between the harvest rate of small pelagic fish and the biomass of jellyfish, i.e. that increased harvest rates could release jellyfish from competition by small pelagic fish. See Supplementary Information for the estimation of harvest rates.

All pairwise associations (*Y* ~ *X*) were tested using a generalised least squares (GLS) regression model, accounting for 1^st^ order autoregressive processes (temporal autocorrelation) using an autoregressive-moving-average model ARMA,^58^. Firstly, we tested simple pairwise associations within each study-area (*A*) (*m*_*A*_, *Y*_*A*_ ~ α + *βX*_*A*_). Secondly, a series of combined models (*m*_1_ - *m*_3_) were constructed to test pairwise associations across all study-areas (*m*_1_, *Y* ~ α + *βX*), by adding study-area as a fixed effect (*m*_2_, *Y* ~ α + *βX + cA*) and by also adding an interaction term between the predictor variable (*X*) and study-area (*m*_3_, *Y* ~ α + *βX* + *cA* + *dXA*). The Akaike information criterion (AIC) was used to select the best model fit. Due to the large number of possible lag-combinations when testing hypothesis H3 across study-areas, this hypothesis was not analysed using the combined models. The statistical power was calculated for all pairwise associations using the *pwr*.*r*.*test* function^59^ in the statistical software R^60^, based on number of observations (*n*), the linear correlation coefficient (Pearson’s *R*) and significance level (95%)^32^. Sensitivity analyses regarding sufficient sample sizes (*n*) and/or effect sizes (*R*) were performed.

In addition to the single pairwise constructs, we applied a confirmatory factor analysis (CFA) to test hypotheses H2 and H3 together in a combined structural equation model (SEM). This model included the following three associations bringing together hypotheses H2 and H3 in a joint model framework (Supplementary Fig. S3); 1) the combined effect of small pelagic fish and jellyfish predation on crustacean zooplankton biomass, *zooplankton* ~ *pelagic fish* + *jellyfish*; 2) the combined effect of fishing mortality and jellyfish predation on small pelagic fish eggs and juveniles (H3) on small pelagic fish biomass, *pelagic fish* ~ *fishery* + *jellyfish*_*prev*_ (where j*ellyfish*_*prev*_ denote jellyfish biomass in previous (1–3) years); and 3) the hypothesised covariation between small pelagic fish and jellyfish biomass (H2), *pelagic fish* ~~ *jellyfish*. This model was analysed for all areas except the Northern Benguela for which we only had one year of observed jellyfish biomass.

We used the CFA function available in the library *lavaan*^61^ for the statistical software R^60^ to analyse the model. Overall model fit was evaluated using a series of model fit indices presented in Kline^33^; the exact-fit hypothesis test *Chi-Square Test of Model Fit* and the approximate fit indices *Root Mean Square Error of Approximation* (RMSEA), *Standardized Root Mean Square Residual* (SRMR) and the *Comparative fit Index* (CFI). According to Kline^33^, an acceptable model fit should have a χ^2^ < *df* (degrees of freedom) with a *p*-value significantly greater than 0.05, indicating that we cannot reject the hypothesis of a perfect model fit. Further, Browne and Cudeck^34^ suggest that point estimates of RMSEA (ε) and/or upper confidence bounds greater than 0.1 indicate that we cannot reject the hypothesis of poor model fit. With regards to the SRMR and the CFI, Hu and Bentler^35^ suggest an acceptable model fit when CFI ≥ 0.95 *and* SRMR ≤ 0.08, although this criteria has been suggested to be too lenient (see Kline^33^).

## Supplementary information

Supplementary information for “Unclear associations between small pelagic fish and jellyfish in several major marine ecosystems”

## Data Availability

All data used in this study are drawn from previously published articles and are referenced in Supplementary Table S1.
