# Supplementary material for: Unclear associations between small pelagic fish and jellyfish in several major marine ecosystems
Source: Sci Rep. 2019 Feb 28;9:2997. doi: 10.1038/s41598-019-39351-7 (PMC6395749; doi:10.1038/s41598-019-39351-7)
Supplement: Supplementary file 1 — Supplementary information for “Unclear associations between small pelagic fish and jellyfish in several major marine ecosystems” [file 41598_2019_39351_MOESM1_ESM.docx]

Supplementary information for “Unclear associations between small pelagic fish and jellyfish in several major marine ecosystems”

Anders Frugård Opdal, Richard D. Brodeur, Kristin Cieciel, Georgi M. Daskalov, Vesselina Mihneva, James J. Ruzicka, Hans M. Verheye and Dag L. Aksnes

Materials and methods

**Biomass time-series**

Cnidarians and ctenophores were allocated to the functional group jellyfish, while forage fish species considered to share the same pelagic habitat where allocated to the functional group small pelagic fish (Table S1). For three of the studied areas, the functional group jellyfish consisted of a single species making up a large fraction of the sample weights; the Southeastern Bering Sea [> 95 %] ^1^, the Northern California Current [> 99 %] ^2^ and the Northern Benguela [> 99 %] ^3^. The functional group small pelagic fish consists of three or more species (Table S1).

For the Southeastern Bering Sea (Fig. S1a), jellyfish and small pelagic fish were sampled at the same stations during summer in two different programs: the BASIS surface trawl survey 2002-2014 ^4^, and the RACE bottom trawl survey for the period 1982 – 2014 ^1,5^. Although the BASIS survey started in 2002, data on jellyfish biomass were not available before 2004. Crustacean zooplankton biomass was estimated during summer (1960-2006) using a twin NORPAC net frame (333 µm mesh size) hauled vertically from near the bottom to the surface at 1 m s^-1^. A calibrated TSK flowmeter was mounted in the center of each net mouth. Sampling locations were variable before 1995, after which yearly fixed sampling stations were established ^6,7^. Annual biomasses were calculated using concentrations (g m^-3^) converted to g m^-2^ using an average tow (bottom) depth of 70 m (Jeff Napp, NOAA, pers. comm.).

For the Northern California Current (Fig. S1b), small pelagic fish, jellyfish and crustacean zooplankton were sampled tri-annually (May, June and September, 1998-2013) during daytime pelagic surveys at the same stations through a surface pelagic survey off the Washington and Oregon coasts, USA ^8-10^. Crustacean zooplankton were sampled from 100 m depth to the surface using vertically towed ring nets (0.5 m diameter, 200 μm mesh), with a frame-mounted General Oceanics calibrated flowmeter. See Suchman *et al*. ^2^ for detailed field and laboratory methods and Ruzicka *et al*. ^9^ for statistical methods used to estimate regional biomasses for log-normally distributed pelagic survey data.

For the Black Sea (Fig. S1c), small pelagic fish biomass was estimated from virtual population analysis based on catch and survey data ^11,12^. Stations for both crustacean and gelatinous zooplankton 1965-2010; ^11^ were sampled on a monthly basis in the western, northern and northeastern Black Sea by two different survey programs: the YuGNIRO (1959-2005), and the IFR (1959-2010).

Crustacean zooplankton were sampled using two different vertically towed plankton nets: a Juday net (37 cm diameter, 150 µm mesh size) and a Bogorov - Rass net (80/113 cm diameter, 500/300 µm mesh size). Until 1988 jellyfish (*Aurelia aurita*) were sampled with both a specially designed 23 m pelagic trawl with an opening of 5 m combined with a vertically towed (0-100 m) Bogorov - Rass net to resolve depth distribution. Filtered water volume was calculated using net mouth area and towed distance. After 1988 jellyfish (*A. aurita* and *Mnemiopsis leidyi*) were sampled using obliquely towed Bongo nets (61 cm diameter, 500/300 µm mesh size) in addition to a vertically towed Bogorov - Rass net (with a frame- mounted flowmeter), which allowed for calibration with pre-1988 sampling. See Kovalev *et al*. ^13^ for a detailed description.

For the Northern Benguela Current (Fig. S1d), small pelagic fish biomass 1961-2000; ^14,15,16^ was also estimated from catch and survey data ^17^, while jellyfish biomass (only year 2003) was estimated from a single acoustic/trawl survey ^18^. Crustacean zooplankton (1978-2010) were collected monthly along a transect off Walvis Bay (23°S) during the SWAPELS program 1978-1989; ^19^, usually using obliquely towed paired Bongo nets (0.25 m^2^ mouth area, 300 μm mesh, 50-0 m) and occasionally a vertically hauled N50V net (0.196 m^2^ mouth area, 80 μm mesh, 50-0 m; ^20^) and during the Monthly Oceanographic Monitoring (2000-2010) program ^21^ using a vertically hauled WP2 net (0.25 m^2^ mouth opening, 200 μm mesh, 200-0 m), and along two transects north and south of Walvis Bay in 1997 ^22^ using obliquely towed paired Bongo nets (0.25 m^2^ mouth area, 300 μm mesh, 200-0 m). During each program, calibrated digital flowmeters were used to measure the volume of water filtered through the nets used for sample collection. Methodological differences between these sampling programs were accounted for according to Verheye *et al*. ^23^ so that the three datasets could be combined to reconstruct a single time-series dataset ^24^. In addition, abundances of cyclopoid copepods (*Oithona* spp.) from the 300-μm meshed net collections were multiplied by 1.85 to make them comparable with those in 200-μm meshed net collections ^25^. Biomass of crustacean zooplankton was estimated based on species- and stage-specific abundances (No. m^-2^) in the upper 200 m, multiplied by their respective individual body masses ^24^.

**Harvest rates**

Harvest rates (fraction of population biomass removed by the fishery per year) of small pelagic fish were estimated based on a series of published sources. For the Southeastern Bering Sea, most of the small pelagic fish biomass consist of species or age groups of species that are not harvested directly, including capelin, smelt and juveniles of cod and pollock. However, there is a directed fishery for spawning herring, and annual estimates of the harvest rates in three main areas of the herring fishery are available from the Alaska Department for Fish and Game; the Bristol Bay area ^26-28^, the Kuskowim area ^29^ and the Alaskan Peninsula – Alutian Islands ^30^. This allowed us to estimate the overall annual harvest rate of small pelagic fish based on the fraction of herring biomass in each of the three areas and the relative fraction of herring in the total biomass of small pelagic fish. Harvest rates for the Northern California Current were based on the annual exploitation rate for the U.S.A. (Northern) component of the Pacific sardine population ^31^, scaled to the annual fraction of sardine in the small pelagic fish biomass. For the Black Sea, total catches of sprat, anchovy and whiting are used ^32,33^, while for the Northern Benguela harvest rate was based on the catches of horse mackerel ^15^ and sardine ^14^.

Tables and figures

| **Table S1.** Time-periods and the most abundant species used to estimate biomass. Combined with mean individual weights, estimated respiration rates and production, these data were used to estimate and compare energy consumption rates of small pelagic fish and jellyfish in four different ecosystems. | | | | |
| --- | --- | --- | --- | --- |
| **Description**  **and area** | **For pelagic fish** | | **For jellyfish** | |
|  | **Value or type** | **Reference** | **Value or type** | **Reference** |
| **Time period** |  |  |  |  |
| Bering Sea |  |  |  |  |
| BASIS survey | 2002-2014 | 4 | 2004-2014 | 4 |
| RACE survey | 1982-2012 | 1, ext. | 1982-2012 | 5, ext. |
| NCC | 1998-2013 | 8, 9 | 1999-2013 | 10 |
| Black Sea | 1960-2010 | 11 | 1965-2010 | 11 |
| N. Benguela | 1960-2007 | 14,15, 16 | 2003 | 18 |
| **Most abundant species** |  |  |  |  |
| Bering Sea | herring, capelin, smelt, juveniles of cod and pollock | 39 | *Chrysaora melanaster* | 5 |
| NCC | sardine, anchovy, herring | 2 | *Chrysaora fuscescens* | 2 |
| Black Sea | anchovy, sprat, horse-mackerel | 11 | *Aurelia aurita, Mnemiopsis leidyi* | 11 |
| N. Benguela | sardine, horse-mackerel | 16 | *Aequorea forskalea* | 18 |

| **Table S2.** Power analyses related to the associations presented in table 2 and figure 2 in the main text. Statistical power was estimated using the pwr.r.test function ^40^, in the statistical software R ^35^ based on number of observations (*n*), the estimated effect size (Pearson’s *R*) and significance level (95%) ^41^. The effect of jellyfish on small pelagic fish recruitment, was analysed with a 1-3 year lag of fish biomass (see Methods). For the Southeastern Bering Sea, two independent surveys are available: a bottom trawl survey (RACE, 1982-2012) and a surface trawl survey (BASIS, 2004-2009). Significant correlations (*p*-value < 0.05) after correcting for first order autoregressive processes ^42^ are denoted in bold. | | | | | | | | | | | | | | |
| --- | --- | --- | --- | --- | --- | --- | --- | --- | --- | --- | --- | --- | --- | --- |
| **Associations**  **and system** | ***pelagic fish~***  ***jellyfish*** | | | **with 1-3 year time-lag**  **(best negative fit shown)** | | | | ***zooplankton~***  ***jellyfish*** | | | ***zooplankton~***  ***pelagic fish*** | | | |
|  | *n* | *R* | *p*ower | *n* | *lag* | *R* | *p*ower | *n* | *R* | *p*ower | *n* | *R* | *p*ower | |
| **Bering Sea** |  |  |  |  |  |  |  |  |  |  |  |  | |  |
| BASIS 2002- | 10 | 0.21 | 0.09 | 8 | 1 | 0.24 | 0.09 | 6 | 0.39 | 0.12 | 8 | 0.61 | | 0.38 |
| RACE 1982- | 31 | 0.15 | 0.13 | 28 | 2 | 0.24 | 0.25 | 28 | 0.21 | 0.22 | 28 | 0.01 | | 0.05 |
| **NCC** | 15 | 0.16 | 0.09 | 14 | 1 | 0.16 | 0.08 | 13 | 0.23 | 0.12 | 14 | 0.27 | | 0.16 |
| **Black Sea** | 46 | 0.07 | 0.08 | 43 | 3 | 0.10 | 0.10 | 46 | **0.57** | 0.99 | 51 | 0.09 | | 0.09 |
| **N. Benguela** |  |  |  |  |  |  |  |  |  |  | 19 | 0.18 | | 0.11 |

| **Table S3.** Structural equation model fit evaluation for three ecosystems, including the exact-fit test *Chi-Square Test of Model Fit* (Chi-square) and the approximate fit indices *Root Mean Square Error of Approximation* (RMSEA), *Standardized Root Mean Square Residual* (SRMR) and the *Comparative Fit Index* (CFI). Model structure is shown in figure S3a. *df* = degrees of freedom and NCC = Northern California Current. | | | | | | | | | |
| --- | --- | --- | --- | --- | --- | --- | --- | --- | --- |
| **Fit indices and system** | **Sample size** | **Chi-square, χ^2^** | | | **RMSEA, ε** | | | **SRMR** | **CFI** |
|  | *n* | *df* | χ^2^ | *p*-value | ε | 90% CI | *p*-value | *estimate* | *estimate* |
| **Bering Sea** | 26 | 4 | 14.4 | 0.006 | 0.317 | 0.151 – 0.500 | 0.009 | 0.193 | 0.00 |
| **NCC** | 12 | 4 | 2.36 | 0.670 | 0.000 | 0.000 – 0.340 | 0.682 | 0.089 | 1.00 |
| **Black Sea** | 43 | 4 | 29.0 | 0.000 | 0.381 | 0.258 – 0.517 | 0.000 | 0.258 | 0.439 |

| 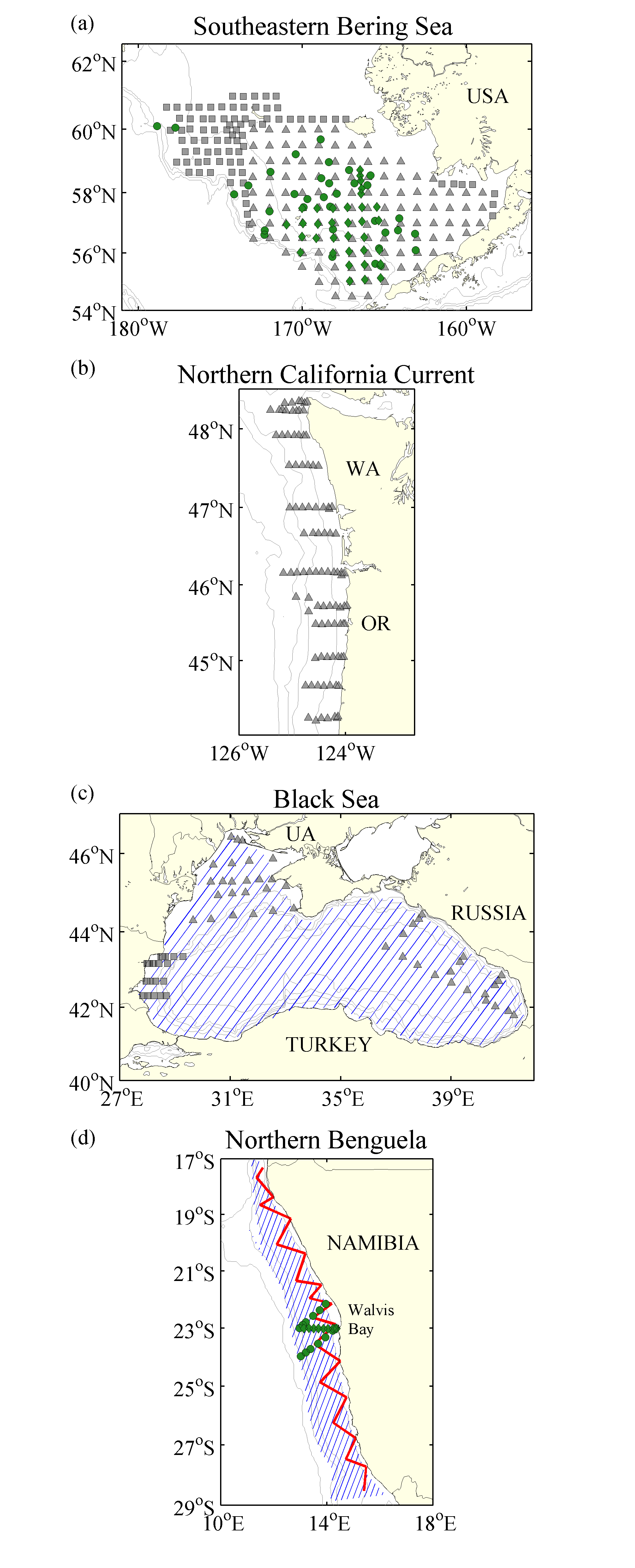 | **Figure S1.** Coverage of survey areas and sampling stations for jellyfish, small pelagic fish and crustacean zooplankton. For the Southeastern Bering Sea (a), jellyfish and small pelagic fish are sampled at the same stations during summer in two different programs: the BASIS surface trawl survey (only grey triangles) 2002-2014 ^4^, and the RACE bottom trawl survey (grey squares and triangles) for the period 1982 – 2014 ^1,5^. For crustacean zooplankton (green), yearly fixed sampling stations between 1995 and 2006 are denoted with green circles, while green diamonds are examples of shelf sampling stations in 1993 and 1963 ^6,7^. For the Northern California Current (b), small pelagic fish, jellyfish and crustacean zooplankton were sampled tri-annually (May, June and September, 1998-2013) at the same stations (triangles) through the pelagic trawl survey ^8-10^. For the Black Sea (c), small pelagic fish biomass was estimated from catch and survey data ^11^ within the blue hatched area ^12^. Stations for both crustacean and gelatinous zooplankton 1965-2010; ^11^ were sampled on a monthly basis by two different survey programs: the YuGNIRO (1959-2005, triangles), and the IFR (1959-2010, squares. For the Northern Benguela Current (d), small pelagic fish biomass 1961-2000; ^14,15,16^ was estimated from catch and survey data within the blue hatched area ^17^, while jellyfish biomass (only year 2003) was estimated from a single acoustic/trawl survey along the red line ^18^. Sampling stations for crustacean zooplankton 1978-2010; ^24^ are denoted by green markers. Triangles represent stations sampled during the SWAPELS program (1978-1989) and the Monthly Oceanographic Monitoring (2000-2010) program ^21^ along the Walvis Bay transect, while circles indicate sampling stations for the year 1997 ^22^. |
| --- | --- |

| 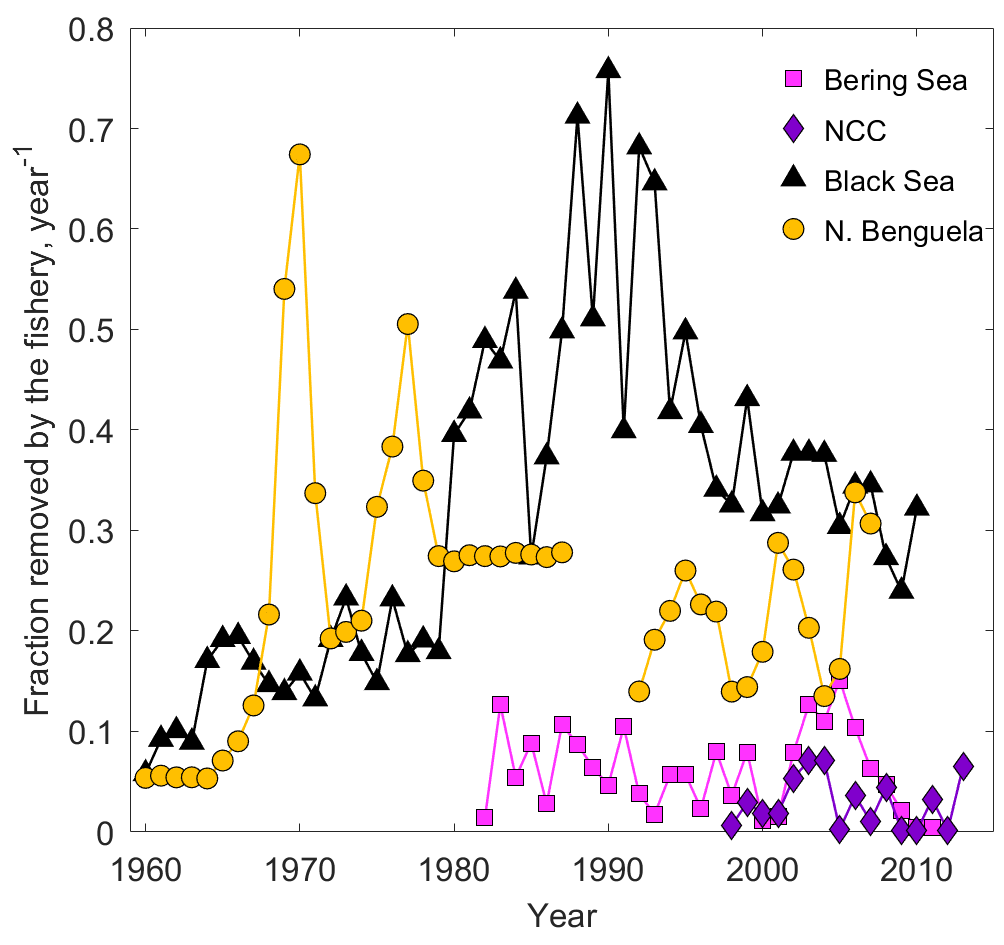 | **Figure S2**. Harvest rates (fraction of population biomass removed by the fishery each year) of small pelagic fish in four ecosystems. For the Southeastern Bering Sea (squares), harvest rates are estimated based on the fishery for spawning herring. Other species and age groups in the Bering Sea (smelt, capelin and juveniles of cod and pollock), are not targeted by the fishery. For the Northern California Current (NCC, diamonds), harvest rate is based on the sardine fishery and the annual sardine fraction in the total small pelagic fish biomass. For the Black Sea (triangles), harvest includes all small pelagic fisheries (anchovy, sprat and horse mackerel), and in the Northern Benguela (circles) harvest rate includes both the sardine and horse mackerel fishery. |
| --- | --- |

| 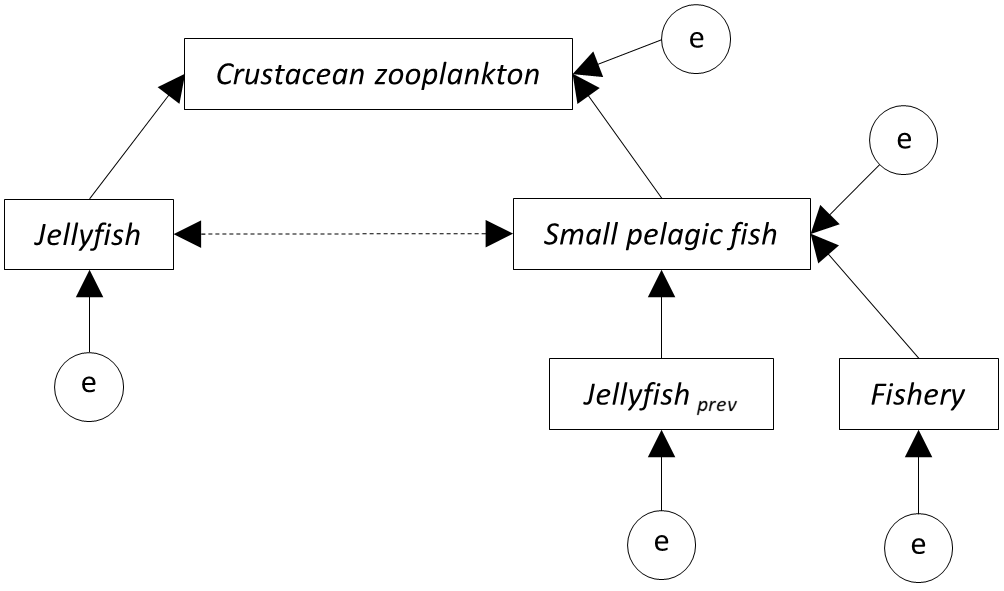 | **Figure S3**. Setup of the structural equation model used to test hypotheses H2 and H3 related to associations between biomass of jellyfish, small pelagic fish and crustacean zooplankton. Effect of fishery is also included. *Jellyfish_prev_* denote jellyfish biomass in previous (1-3) years, hypothesised (H3) to influence small pelagic fish recruitment and consequently small pelagic fish biomass. Error terms are denoted with *e*, and whole line arrows denote first order direction of causality. Dotted lined arrow suggest expected covariation. |
| --- | --- |
|  |  |

References

1 Brodeur, R. D., Sugisaki, H. & Hunt, G. L. Increases in jellyfish biomass in the Bering Sea: implications for the ecosystem. *Marine Ecology Progress Series* **233**, 89-103, doi:10.3354/meps233089 (2002).

2 Suchman, C. L., Brodeur, R. D., Daly, E. A. & Emmett, R. L. Large medusae in surface waters of the Northern California Current: variability in relation to environmental conditions. *Hydrobiologia* **690**, 113-125, doi:10.1007/s10750-012-1055-7 (2012).

3 Lynam, C. P., Heath, M. R., Hay, S. J. & Brierley, A. S. Evidence for impacts by jellyfish on North Sea herring recruitment. *Marine Ecology Progress Series* **298**, 157-167, doi:10.3354/meps298157 (2005).

4 Decker, M. B. *et al.* Jellyfish and forage fish spatial overlap on the eastern Bering Sea shelf during periods of high and low jellyfish biomass. *Marine Ecology Progress Series* **591**, 57-69, doi:10.3354/meps12273 (2018).

5 Brodeur, R. D. *et al.* Rise and fall of jellyfish in the eastern Bering Sea in relation to climate regime shifts. *Progress in Oceanography* **77**, 103-111, doi:10.1016/j.pocean.2008.03.017 (2008).

6 Stabeno, P. J. *et al.* Comparison of warm and cold years on the southeastern Bering Sea shelf and some implications for the ecosystem. *Deep-Sea Research Part II-Topical Studies in Oceanography* **65-70**, 31-45, doi:10.1016/j.dsr2.2012.02.020 (2012).

7 Napp, J. M. *et al.* Interannual and decadal variability in zooplankton communities of the southeast Bering Sea shelf. *Deep-Sea Research Part II-Topical Studies in Oceanography* **49**, 5991-6008, doi:10.1016/s0967-0645(02)00330-2 (2002).

8 Brodeur, R. D., Fisher, J. P., Emmett, R. L., Morgan, C. A. & Casillas, E. Species composition and community structure of pelagic nekton off Oregon and Washington under variable oceanographic conditions. *Marine Ecology Progress Series* **298**, 41-57, doi:10.3354/meps298041 (2005).

9 Ruzicka, J. J. *et al.* Interannual variability in the Northern California Current food web structure: Changes in energy flow pathways and the role of forage fish, euphausiids, and jellyfish. *Progress in Oceanography* **102**, 19-41, doi:10.1016/j.pocean.2012.02.002 (2012).

10 Ruzicka, J. J., Daly, E. A. & Brodeur, R. D. Evidence that summer jellyfish blooms impact Pacific Northwest salmon production. *Ecosphere* **7**, (doi: 10.1002/ecs1002.1324), doi:10.1002/ecs2.1324 (2016).

11 Daskalov, G. M. *et al.* Architecture of collapse: regime shift and recovery in an hierarchically structured marine ecosystem *Global Change Biology* **23**, 1486-1498, doi:doi:10.1111/gcb.13508 (2017).

12 FAO. The State of World Fisheries and Aquaculture 2014. *Food and Agriculture Organization of the United Nations. Rome, Italy*, 223 pp (2014).

13 Kovalev, A. *et al.* Long-term changes in the Black Sea zooplankton: the role of natural and anthropogenic factors. *NATO Science Series Partnership Sub-Series 2 Environmental Security* **47**, 221-234 (1998).

14 Kirchner, C. H., Bartholomae, C. H. & Kreiner, A. Use of environmental parameters to explain the variability in spawner-recruitment relationships of Namibian sardine *Sardinops sagax*. *African Journal of Marine Science* **31**, 157-170, doi:10.2989/ajms.2009.31.2.4.876 (2009).

15 Mundjulu, I. E. *Trends in the fishery and population traits of the Namibian stock of Cape horse mackerel Trachurus trachurus capensis (Castelnau, 1861)* PhD thesis, Universtity of Tromsø, (2009).

16 Boyer, D. C. & Hampton, I. An overview of the living marine resources of Namibia. *South African Journal of Marine Science* **23**, 5-35 (2001).

17 Flynn, B. A. *et al.* Temporal and spatial patterns in the abundance of jellyfish in the northern Benguela upwelling ecosystem and their link to thwarted pelagic fishery recovery. *African Journal of Marine Science* **34**, 131-146, doi:10.2989/1814232x.2012.675122 (2012).

18 Lynam, C. P. *et al.* Jellyfish overtake fish in a heavily fished ecosystem. *Current Biology* **16**, 492-493 (2006).

19 Shannon, L. V. & Pillar, S. C. in *Oceanography and Marine Biology: An Annual Review* (ed M. Barnes) 65-170 (University Press, 1986).

20 Kemp, S., Hardy, A. C. & Mackintosch, N. A. Objects, equipment, and methods. *Discovery Reports* **1**, 143-232 (1929).

21 Kreiner, A. & Yemane, D. Spatial and temporal variability of copepod communities along a monitoring transect in the Northern Benguela from 2000 to 2010 in relation to environmental parameters. *In: Annual Research Meeting: “From monitoring to Science”, Ministry for Fisheries and Marine Resources, Swakopmund, 262-276* (2013).

22 Rippe, L. & Téjédor, M. F. Mesozooplankton community structure and standing stock off Central Namibia during early upwelling season (unpublished manuscript). (1999).

23 Verheye, H. M., Richardson, A. J., Hutchings, L., Marska, G. & Gianakouras, D. Long-term trends in the abundance and community structure of coastal zooplankton in the southern Benguela system, 1951-1996. *South African Journal of Marine Science* **19**, 317-332 (1998).

24 Verheye, H. M., Lamont, T., Huggett, J. A., Kreiner, A. & Hampton, I. Plankton productivity of the Benguela Current Large Marine Ecosystem (BCLME). *Environmental Development* **17**, 75-92 (2016).

25 Pillar, S. C. A comparison of the performance of four zooplankton samplers. *South African Journal of Marine Science* **2**, 1-18, doi:DOI: 10.2989/02577618409504354 (1984).

26 Sands, T. *et al.* 2007 Bristol Bay area annual management report. Fishery Management Report No. 08-28. 1-145 (Alaska Department of Fish and Game, Anchorage, Alaska, 2008).

27 Skrade, J. R. *et al.* 1987 Bristol Bay annual management report. 1-294 (Alaska Department of Fish and Game, Anchorage, Alaska, 1988).

28 Jones, M. *et al.* 2015 Bristol Bay Area Annual Management Report. Fishery Management Report No. 16-13. (Alaska Department of Fish and Game, Anchorage, Alaska, 2016).

29 Poetter, A. D., Tiernan, A. & Lipka, C. Annual management report Kuskokwim area, 2015. Fishery Management Report No. 16-38. 1-113 (Alaska Department for Fish and Game, 2016).

30 Lipka, C. Memorandum: Summary of the 2016 Alaska Peninsula-Alutian Islands Herring (Alaska Department of Fish and Game, 2017).

31 Hill, K. T. *et al.* Assessment of the Pacific sardine resource in 2014 for U.S.A. management in 2014-15. 1-182 (Southwest Fisheries Science Center. NOAA National Marine Fisheries Service, La Jolla, California, 2014).

32 Prodanov, K. *et al.* Environmental management of fish resources in the Black Sea and their rational exploitation. *Studies and Reviews. General Fisheries Council for the Mediterranean* **68**, 1-178 (1997).

33 STECF. 2013 Assessment of Black Sea stocks. *Scientific, Technical and Economic Committee for Fisheries (STECF)* **EWG 13-20**, 1-429, doi:10.2788/34535 (2013).

34 Rosseel, Y. lavaan: An R Package for Structural Equation Modeling. *Journal of Statistical Software* **48**, 1-36, doi:http://www.jstatsoft.org/v48/i02/ (2012).

35 R Core Team. *R: A language and environment for statistical computing*. (R Foundation for Statistical Computing, 2013).

36 Kline, R. B. *Principles and practice of structural equation modeling*. 3 edn, (The Guilford Press, 2011).

37 Browne, M. W. & Cudeck, R. in *Testing structural equation models* (eds K. A. Bolllen & J. S. Long) 136-162 (Sage, 1993).

38 Hu, L. T. & Bentler, P. M. Cutoff Criteria for Fit Indexes in Covariance Structure Analysis: Conventional Criteria Versus New Alternatives. *Structural Equation Modeling-a Multidisciplinary Journal* **6**, 1-55, doi:10.1080/10705519909540118 (1999).

39 Brodeur, R. D., Wilson, M. T., Walters, G. E. & Melnikov, I. V. in *Dynamics of the Bering Sea: A Summary of Physical, Chemical, and Biological Characteristics, and a Synopsis of Research on the Bering Sea* (eds T.R. Loughlin & K. Ohtani) 509–536 (University of Alaska Sea Grant, 1999).

40 Champely, S. *et al.* pwr: Basic Functions for Power Analysis. *R package version 1.2-2* (2018).

41 Cohen, J. *Statistical power analysis for the behavioral sciences* (Lawrence Erlbaum, 1988).

42 Pyper, B. J. & Peterman, R. M. Comparison of methods to account for autocorrelation in correlation analyses of fish data. *Canadian Journal of Fisheries and Aquatic Sciences* **55**, 2127-2140 (1998).
